# A Pilot Study Evaluating the Effects of Magtrace® for Sentinel Node Biopsy in Breast Cancer Patients Regarding Care Process Optimization, Reimbursement, Surgical Time, and Patient Comfort Compared With Standard Technetium^99^

**DOI:** 10.1245/s10434-020-09280-1

**Published:** 2020-12-01

**Authors:** Sina Shams, Kai Lippold, Jens Uwe Blohmer, Robert Röhle, Friedrich Kühn, Maria Margarete Karsten

**Affiliations:** 1 Department of Gynecology with Breast Center, Charité – Universitätsmedizin Berlin, Corporate Member of Freie Universität Berlin, Humboldt-Universität zu Berlin and Berlin Institute of Health, Berlin, Germany; 2 Directorate of Charité Center 17, Charité – Universitätsmedizin Berlin, Corporate Member of Freie Universität Berlin, Humboldt-Universität zu Berlin and Berlin Institute of Health, Berlin, Germany; 3 Institute of Biometry and Clinical Epidemiology, Charité – Universitätsmedizin Berlin, Corporate Member of Freie Universität Berlin, Humboldt-Universität zu Berlin and Berlin Institute of Health, Berlin, Germany

## Abstract

**Background:**

Sentinel lymph node biopsy after technetium-99 (Tc^99^) localization is a mainstay of oncologic breast surgery. The timing of Tc^99^ injection can complicate operating room schedules, which can cause increasing overall costs of care and patient discomfort.

**Methods:**

This study compared 59 patients who underwent breast cancer surgery including sentinel lymph node biopsy. Based on the surgeon’s choice, 29 patients were treated with Tc^99^, and 30 patients received the iron-based tracer, Magtrace. The primary outcomes were time spent on the care pathway and operating time from commissioning of the probe to removal of the sentinel node. The secondary outcomes were patient pain levels and reimbursement.

**Results:**

The mean time spent on the preoperative breast cancer care pathway was significantly shorter for the Magtrace group (5.4 ± 1.3 min) than for the Tc^99^ group (82 ± 20 min) (*p* < 0.0001). The median time from probe usage to sentinel node extirpation was slightly but not significantly shorter in the Magtrace group (5 min; interquartile range [IQR], 3–15 min vs 10 min; IQR, 7–15 min; *p* = 0.151). Reimbursement and pain levels remained unchanged, and the hospital length of stay was similar in the two groups (Magtrace: 5.1 ± 2.3 days vs Tc^99^: 4.5 ± 3.2 days).

**Conclusions:**

Magtrace localization shortened the preoperative care pathway and did not affect surgical time or reimbursement. Once established, it could allow for cost reduction and improve patient comfort.

Sentinel node biopsy (SNB) is an integral part of diagnosis and therapy of early breast cancer. Not only the therapy regimen but also the prognosis of patients is highly influenced by the nodal status.^1^ In recent years, SNB has replaced axillary lymph node dissection (ALND) as a staging procedure for patients with clinically negative lymph nodes (cN0) because it allows determination of the nodal status and reduces the burden often caused by full ALND.^2^ Consequently, replacement of ALND by SNB has led to an increase in the quality of life for women who undergo breast cancer treatment.3,^4^

The SNB procedure can be performed in several ways depending on the tracer used for localization. The most widely used tracer is technetium-99 (Tc^99^) and sometimes blue dye if dual tracers^5^ are needed or Tc^99^ is not available. The use of Tc^99^, however, comes with certain disadvantages.^6^ In addition to the effect of its radioactivity on patients and medical personnel, clinicians must consider the special handling of the tracer, the additional training of staff, and the specific regulatory requirements. Due to its short half-life (~ 6 h) and dependency on the Department of Nuclear Medicine, the use of Tc^99^ also causes logistical restrictions, particularly concerning operating room scheduling.

Alternatives that allow for more flexible scheduling and work without radioactivity have been developed recently with the idea to improve the care pathway and thus patient comfort as well as clinical workflow. Available lymph node tracers that fall into this category are indocyanine green (ICG) and superparamagnetic iron oxide (SPIO). Both of these tracers have proven to be equivalent to the conventional radioisotope, Tc^99^, in terms of detection rates, false-negative rates, and safety.^7^^–^13 However, ICG has not been approved by the European Medicines Agency (EMA) or the U.S. Food and Drug Administration (FDA) for SNB as a part of breast cancer treatment and may be used only off-label in clinical trials.^14^ In contrast, SPIO (e.g., Magtrace; Endomagnetics, Cambridge, UK) is already CE (Conformité Européenne)- and FDA-approved for sentinel node localization and used in clinical practice.

The SPIO tracer is a rust-colored solution containing an iron oxide compound coated with carboxydextran that can be applied in a timeframe ranging from 20 min to 7 days before surgery. During surgery, SPIO is located with a handheld magnetometer (e.g., Sentimag; Endomagnetics) similar to the conventional gamma probe. In most cases, especially when SPIO is injected 1 to 3 days before surgery, lymph nodes are stained brownish, supporting the localization visually.^15^ With the use of SPIO, the problems associated with Tc^99^ could be significantly reduced or, in the case of radiation exposure, avoided. At the same time, SPIO might allow for a much easier scheduling of presurgical procedures, reducing patient anxiety and risk for delays in the care pathway.^16^^–^18

To our knowledge, the effect of using Magtrace instead of Tc^99^ on the clinical workflow and treatment costs has not been investigated to date, and publications on the patient’s perspective are rare. This study therefore aimed to evaluate the impact of Magtrace on the pre- and intraoperative care pathway and patient well-being, as well as its effect on reimbursement in the German health care system.

## Methods

### Study Design

Between May 2019 and January 2020, female patients who underwent breast surgery (breast-conserving surgery or mastectomy) and SNB for invasive breast cancer (cT1 to cT3, cN0, cM0) at the breast center of Charité—Universitätsmedizin Berlin were offered inclusion into the trial.

The exclusion criteria ruled out patients younger than 40 years, BRCA1/2 mutation, breast composition level C or higher according to the fifth edition of the American College of Radiology Breast Imaging-Reporting and Data System (ACR BI-RADS),^19^ high likelihood of a breast magnetic resonance imaging (MRI) in the next 5 years, hypersensitivity to iron oxide or dextran compounds, hemochromatosis, and metal implants in the axilla or chest.

**Fig. 1:**
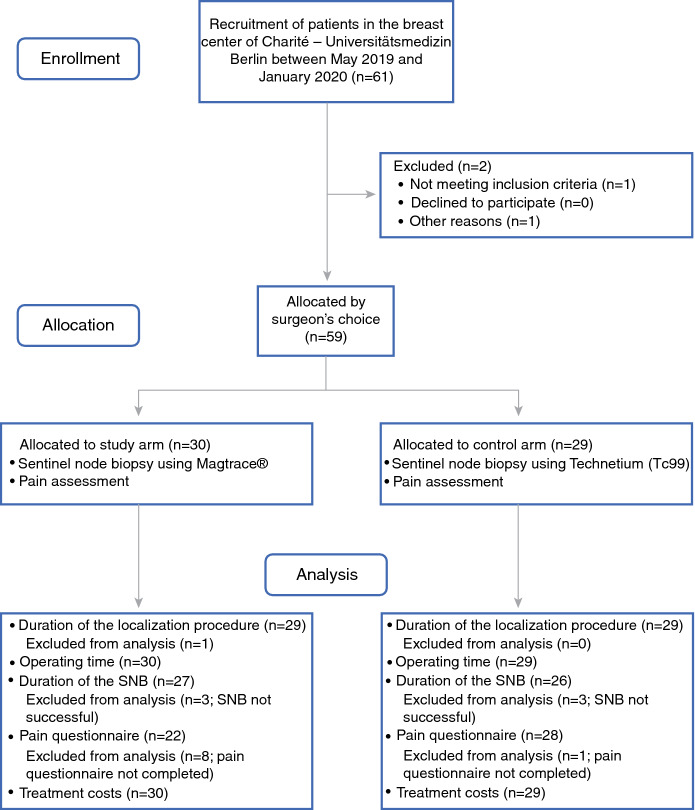
Group allocation and course of the study. SNB, sentinel node biopsy

The 59 patients enrolled in the study were equally allocated to the study arm (sentinel node localization using Magtrace) and the control arm (conventional sentinel node localization using Tc^99^). The group allocation was by the surgeon’s choice, as illustrated in Fig. 1.

The study was approved by the institutional review board of Charité—Universitätsmedizin Berlin, and all the participating patients gave written informed consent.

### Outcomes

The primary outcomes of the study were time spent on the care pathway, total duration of the sentinel lymph node localization procedure, and operating time from commissioning of the probe until sentinel extraction.

The secondary outcomes were patient pain levels and overall treatment costs.

### Tracer Injection

The patients in the control group received sentinel lymph node localization by subcutaneous injection of 40 or 180 MBq of Tc^99^ (Tc-99 m-NanoHSA; Rotop, Dresden, Saxony, Germany) at four periareolar injection sites. The procedure was performed in the Department of Nuclear Medicine. Lymphoscintigraphy, following our internal policy, had to be performed after the injection in all cases. We started time measurement as soon as the patients left the ward or outpatient clinic for this procedure and stopped it when they returned. In all cases, the time for the patient’s way to the nuclear medicine department and back, the waiting time before and after the tracer injection, and the times for preparation, Tc^99^ administration, and subsequent lymphoscintigraphy were all measured separately. For 17 patients, Tc^99^ was applied on the day of surgery, and for 12 patients, it was applied 1 day earlier.

During their preoperative outpatient clinic visit, the patients in the study group received 2 ml of SPIO (Magtrace) as an interstitial perimammillary injection in the outer upper quadrant of the affected breast. The injection was administered 3 days for 5 patients and 1 day before surgery for 23 patients. In two cases, it was administered intraoperatively. The time was measured, starting when the patients were undressing and ending when they finished redressing. If the tracer was injected in the operating room, time measurement began as soon as the preparations for the injection started and stopped with removal of the needle.

### Pain Assessment

All the patients were asked to complete QUIPS (Qualitätsverbesserung in der postoperativen Schmerztherapie/translation: quality improvement in postoperative pain management), the pain questionnaire, before and immediately after injection of the tracer. This widely known and verified German pain questionnaire consists of 16 questions with numeric rating scales (0–10) to assess the patient’s pain level.^20^ It asks not only for acute pain levels but also for pain-related limitations and pain therapy.

### Surgery

All but one patient underwent either breast-conserving surgery or mastectomy. In one case, the patient received SNB only. Five different surgeons participated in the trial.

Intraoperatively, we first checked the detectability of both tracers by scanning the injection site. The radioactive tracer was detected with the Gamma Finder II (WOM [World of Medicine] GmbH, Berlin, Germany) and the magnetic tracer with the SentiMag Gen2 (Endomagnetics, Cambridge, UK). In all 59 cases, this test was positive.

We performed SNB through the breast incision in 31 cases (Magtrace: 19; Tc^99^: 12) and through an additional axillary incision in 28 cases (Magtrace: 11; Tc^99^: 17). Time measurement of the SNB began with the first and definite use of the probe and ended with extraction of the sentinel lymph node.

The lymph node with the highest signal was considered the sentinel lymph node. After extraction, we verified the signal ex vivo and checked the axilla for remaining signals. We continued the axillary surgery if a remaining signal was at least 10% higher than the signal of the initially retrieved lymph node. In nine cases of the SPIO group, we extracted more than one lymph node, whereas in all 29 cases of the control group, only one lymph node had to be removed.

### Statistical Analysis

The sample size was calculated with SPSS SamplePower (IBM, Armonk, New York, NY, USA) using a two-sample *t* test with the hypothesized duration of the preoperative localization procedures. We estimated 27.2 min for the preoperative pathway using Magtrace and 38.1 min using Tc^99^. Assuming that the data were distributed normally, we calculated a sample size of 28 patients per group.

Data concerning clinical and pathologic features were statistically analyzed using SPSS version 26.0 (IBM). The patients who underwent bilateral surgery were considered as two cases for all features except age and BMI because the treatments of the affected breasts were considered sufficiently independent of each other. Thereby, the time for each injection was measured separately, and the time for undressing and redressing or for going to the nuclear medicine department and back was counted twice.

Kolmogorov–Smirnov and Shapiro–Wilk tests of normality were performed, and the values are presented as median (interquartile range, [IQR]) or as mean ± standard deviation. Depending on data distribution, the Mann–Whitney *U* test or the two-sample *t* test was applied for continuous variables. Categorical variables were compared using the Chi square-test or Fisher’s exact test. A *p* value lower than 0.05 was considered statistically significant.

### Economic Analysis

All the patients in the Tc^99^ and Magtrace groups received their breast cancer surgery during an inpatient visit with overnight hospital admission. In Germany, all inpatient treatment is reimbursed through the flat-rate-based diagnosis-related group (DRG) system. To evaluate the impact of the different localization methods, we compared the final DRGs in both arms. All cases were grouped according to the German DRG 2019 system, including cases that had been admitted in 2020.

## Results

### Patient Cohort

The analysis included 59 patients. Of these patients, 29 received SNB using Tc^99^ and 30 received SNB using Magtrace.

An overview of the demographics as well as the clinical and histopathologic features of the cohort is shown in Table 1.

**Table 1:** Clinical and histopathologic features of the patient cohort

	Magtrace	Technetium-99	*P* Value^a^
	(*n* = 30)	(*n* = 29)	
	*N* (%)	*N* (%)	
Age (years)			0.594^b^
Mean ± SD	60.9 ± 11.1	62.6 ± 13.3	
BMI (kg/m^2^)			**0.028**^c^
Median (IQR)	24.8 (22.9–29.3)	23.1 (20.7–25.5)	
Missing	1	0	
Lateralization^f^			0.301^e^
Left	17 (56.6)	12 (41.4)	
Right	13 (43.4)	17 (58.6)	
Tumor stage			0.904^d^
pTis	1 (3.3)	0 (0)	
pT0	1 (3.3)	2 (6.9)	
pT1	13 (43.3)	11 (37.9)	
pT2	13 (43.3)	13 (44.8)	
pT3	2 (6.7)	3 (10.3)	
Missing	0	0	
Nodal stage			0.761^d^
pN0	22 (73.3)	21 (72.4)	
pN1	8 (26.7)	6 (20.7)	
pN2	0 (0)	1 (3.4)	
pNX	0 (0)	1 (3.4)	
Missing	0	0	
Grade			0.077^d^
G1	6 (22.2)	1 (3.8)	
G2	19 (70.4)	19 (73.1)	
G3	2 (7.4)	6 (23.1)	
Missing^g^	3	3	
Accompanying DCIS	9 (30)	16 (55.2)	0.067^e^
Histopathologic type			0.091^d^
NST	16 (55.2)	21 (77.8)	
ILC	9 (31)	2 (7.4)	
Other	4 (13.8)	4 (14.8)	
Missing	1	2	
ER status			**0.007**^d^
Positive	26 (100)	17 (73.9)	
Negative	0 (0)	6 (26.1)	
Missing	4	6	
HER2 status			0.117^d^
0	8 (32)	7 (30.4)	
0–1	1 (4)	0 (0)	
1+	15 (60)	9 (39.1)	
2+	1 (4)	4 (17.4)	
3+	0 (0)	3 (13)	
Missing	5	6	

SD, standard deviation; BMI, body mass index; IQR, interquartile range; DCIS, ductal carcinoma in situ; NST, invasive carcinoma of no special type; ILC, invasive lobular carcinoma; ER: estrogen receptor (positive if expression ≥ 1%); HER2, human epidermal growth factor receptor 2

^a^Bold *p* values are significant

^b^Two sample *t* test

^c^Mann-Whitney *U* test

^d^Fisher’s exact test

^e^Chi square test

^f^In the Magtrace group, one patient underwent bilateral mastectomy

^g^Grading is missing in a total of six cases: two due to pathologic complete response after neoadjuvant chemotherapy and in two other cases due to neoadjuvant systemic therapy. In the remaining cases, histopathologic analysis resulted in metaplastic breast cancer and DCIS

The mean age at surgery was similar in the two groups (Magtrace: 60.9 ± 11.1 years vs Tc^99^: 62.6 ± 13.3 years; *p* = 0.594). The tumors and nodal stages were evenly distributed and did not differ significantly between the study arm and the control arm. In contrast, the body mass index (BMI) was slightly higher in the Magtrace group (24.8 kg/m^2^; IQR, 22.9–29.3 kg/m^2^) than in the Tc^99^ group (23.1 kg/m^2^; IQR, 20.7–25.5 kg/m^2^; *p* = 0.028). A significant difference was shown concerning the estrogen-receptor expression, with 100% of the tumors estrogen receptor-positive in the Magtrace group compared with only 73.9% of the tumors estrogen receptor-positive in the control group (*p* = 0.007).

### Pre- and Intraoperative Course

Differences between the groups in terms of the pre- and intraoperative course are presented in Table 2. The average time of 5.4 ± 1.3 min spent on the preoperative breast care pathway in the Magtrace group was significantly shorter than the 82 ± 20 min in the Tc^99^ group (*p* < 0.0001). With the time for lymphoscintigraphy excluded, the duration of the preoperative pathway still was significantly longer in the control group (54.4 ± 13.6 min; *p* < 0.0001).

**Table 2:** Pre- and intraoperative course

	Magtrace	Technetium-99	*P* Value^a^
	(*n* = 30) *n* (%)	(*n* = 29) *n* (%)	
Type of surgery			0.158^b^
Breast-conserving surgery	18 (60)	22 (76)	
Mastectomy	12 (40)	6 (21)	
Only SNB	0 (0)	1 (3)	
Successful localization procedure			1.000^b^
Yes	30 (100)	29 (100)	
No	0 (0)	0 (0)	
Duration of the localization procedure (min)			**< 0.0001**^c^
Mean ± SD	5.4 ± 1.3	82 ± 20	
Missing	1	0	
Duration of the localization procedure without lymphoscintigraphy (min)			**< 0.0001**^c^
Mean ± SD	5.4 ± 1.3	54.4 ± 13.6	
Missing	1	0	
Sentinel lymph node detected			1.000^b^
Yes	27 (90)	26 (89.6)	
No	3 (10)	3 (10.3)	
No. of retrieved lymph nodes			**< 0.0001**^d^
Median (Range)	1 (1–7)	1 (1–1)	
Duration of the sentinel extraction (min)^e^			0.151^d^
Median (IQR)	5 (3–15)	10 (7–15)	
Missing	3	3	
Duration of the whole SNB (min) ^f^			0.412^d^
Median (IQR)	9 (4–15)	10 (7–15)	
Missing	3	3	
Brown-dyed sentinel lymph nodes			
Yes	20 (100)		
No	0 (0)		
Missing	10		
Operating time (min)			0.891^d^
Median (IQR)	74 (58–99)	71 (56–87)	
Completed pain questionnaires (QUIPS)	22 (73.3)	28 (96.5)	**0.026**^b^
Not completed	8	1	

SNB, sentinel node biopsy; SD, standard deviation; IQR, interquartile range

^a^Bold *p* values are significant

^b^Fisher’s exact test

^c^Two-sample *t* test

^d^Mann-Whitney *U* test

^e^Time from the first and definite use of the probe until sentinel extraction

^f^Time from the first and definite use of the probe until removal of the last marked lymph node

Intraoperatively, the median time from the first and definite usage of the probe to sentinel extraction was 5 min (IQR, 3–15 min) in the Magtrace group and 10 min (IQR, 7–15 min) in the Tc^99^ group (*p* = 0.151). Consequently, the duration of the SNB was slightly but not significantly shorter when Magtrace was used for sentinel node localization.

The two groups were similar in duration of the whole SNB procedure (i.e., removal of all marked lymph nodes; Magtrace: 9 min [IQR, 4–15 min] vs Tc^99^: 10 min [IQR, 7–15 min]; *p* = 0.412) and overall surgery (Magtrace: 74 min [IQR, 58–99 min] vs Tc^99^: 71 min [IQR, 56–87 min]; *p* = 0.891).

### Pain Levels

Significantly fewer patients in the study arm completed the QUIPS pain questionnaire (Magtrace: 22 [73.3%] vs Tc^99^: 28 [96.5%]; *p* = 0.026).

We could not find any relevant differences between the two groups regarding the pain assessment and pain therapy before and after the localization procedure.

The median pain level after the tracer injection in the Tc^99^ arm was 0 (IQR, 0–1), and all the patients in the Magtrace group reported no pain at all.

### Economic Analysis

*Reimbursement.* Since the introduction of DRGs in 2004, German hospitals receive reimbursement based on the G-DRG system, which is comparable with the Medicare part A reimbursement scheme. Both plans use prospective payment systems in which institutions receive a fixed remuneration per hospital admission for each DRG.^21^ In Germany, patients are grouped into DRGs based on their diagnoses and procedures, with the diagnoses coded according to the International Statistical Classification of Diseases and Related Health Problems (ICD) and the procedures coded according to the Operation and Procedure Code (OPS).^22^ In this regard, the so-called case mix points (CMPs) describe the clinical complexity and the amount of resources required to treat the patient, with higher CMPs representing more complex and challenging cases.^23^ As described earlier, hospitals receive a lump sum for each DRG, which should cover all incurred costs. In theory, surpluses for less complex cases and additional costs for a higher care effort should balance each other out. Hospitals also are reimbursed the full DRG amount only if patients stay in the hospital for a defined period specific for each DRG code.

In our study, the DRGs differed significantly between the two groups, and the mean reimbursement showed higher CMPs in the Magtrace group. However, an in-depth analysis of the underlying OPS and ICD coding proved that the variation in DRGs did not result from the type of sentinel node localization, but rather from differences in the underlying surgical approach. The use of Magtrace did not generate a change in OPS coding, whereas the Tc^99^-based sentinel node localization generated an additional OPS code by the Department of Nuclear Medicine. Therefore, all cases from the Magtrace arm had to be regrouped by addition of the omitted code to detect any changes. However, the regrouping did not lead to any changes in DRGs. Hence, the use of Magtrace did not affect the DRG outcome, and reimbursement remained the same independent of the localization method.

*Impact on the Care Pathway.* The Magtrace group had 9 cases with a preoperative day compared with 12 cases in the Tc^99^ group. The mean hospital length of stay (LoS) was 5.1 ± 2.3 days in the Magtrace group and 4.5 ± 3.2 days in the Tc^99^ group. Due to the variance in DRG outcome, comparison of the LoS had to be made according to the Institut für das Entgeltsystem im Krankenhaus (translation: Institute for payment systems in hospitals) (InEK)-Benchmark defined as the average LoS for treatment of a patient in Germany. Application of the InEK-Benchmark resulted in a mean LoS of 92% in the Magtrace group and 94% in the Tc^99^ group (a LoS of 92% means that patients in our clinic were discharged 8% earlier than the German average 2 years earlier).

## Discussion

Several publications have proven the ability of SPIO to safely and correctly localize the sentinel lymph node, which then can be detected using the Sentimag probe.^11^^,^^13^ Therefore, SPIO-guided SNB can be considered equivalent to the Tc^99^-based approach, which is why we chose to investigate the consequence of using this tracer for the care pathway and reimbursement. According to the “Arbeitsgemeinschaft Gynäkologische Onkologie” (Working Group Gynecological Oncology), an independent community within the German Society of Gynecology and Obstetrics and the German Cancer Society, the use of SPIO for SNB is recommended with a +/- rating. In contrast, SNB with Tc^99^ is rated ++.^24^ This contrast is due to the lack of prospective and randomized studies. However, meta-analyses have proven the efficacy of SPIO for SNB.^8^^,^^13^

Our results showed that iron-based sentinel lymph node localization significantly shortened the time spent on the preoperative care pathway. The overall operating time and pain levels were similar to those of the conventional approach, whereas the time needed for sentinel lymph node extraction was slightly but not significantly shorter in the Magtrace group. The new localization method did not affect the reimbursement, and the average LoS was similar in the two groups.

Scheduling Tc^99^-based SNB is impeded by the tracer’s radioactive half-life (~ 6 h) and availability of nuclear medicine specialists and equipment. With the use of Magtrace, an independent, shortened, and thus simplified preoperative care pathway facilitates operating room scheduling by enabling surgery postponement within a time frame of 7 days after injection. Magtrace can be administered up to 20 min before surgery, which also allows for short-term changes to the operating room schedule. In addition, the large range in time during which Magtrace can be administered ultimately could benefit rural areas lacking access to nuclear medicine facilities. Our Magtrace patients spent only about 5 ± 1.3 min in the preoperative care pathway dedicated to sentinel lymph node marking, whereas the Tc^99^ group required a mean of 82 ± 20 min. These time savings might particularly benefit elderly or weak patients as well as patients with a long journey to the hospital because unnecessary consultations or overnight stays before surgery can be avoided by marking sentinel lymph nodes intraoperatively or during the preoperative outpatient appointment.

Although Magtrace localization is a new surgical method for our institution and staff, our study reported a shorter duration of sentinel lymph node extraction. In accordance with other research groups, we assume a steep learning curve due to the familiar handling of the Sentimag probe,^25^ which is similar to the Gamma Finder used for the detection of Tc^99^.

Surgery delay and complications during same-day preoperative marking procedures could further worsen psychological distress and anxiety.^26^^,^^27^ We did not detect any differences in pain levels, but an increase in general patient comfort is to be expected, nonetheless. With the implementation of iron-based SNB, same-day marking procedures during the preoperative visit will reduce further appointments and imaging (lymphoscintigraphy), thereby improving the patients’ experience. It has been shown that SNB is equally precise even without the information provided by preoperative lymphoscintigraphy.^28^ However, even with omission of the time needed for lymphographic imaging, preoperative SNB preparation took significantly longer when Tc^99^ was used as the lymph node tracer.

Reimbursement was similar in the two groups, but we found that using Magtrace had a cost-saving effect compared with the use of Tc^99^ at our institution. In 2019, the costs for Tc^99^-based lymph node localization at the Charité Department of Nuclear Medicine were €360 per case for medical staff, material, infrastructure, and clinic overhead. This stands in contrast to the costs associated with the time required for Magtrace injection by a senior breast surgeon, which were calculated at €7.50. Acquisition costs for the Magtrace solution and probe have yet to be added, but are a matter of negotiation. Costs might therefore differ from hospital to hospital but are expected to be lower than the previous expenses.

Lack of consistency in the care pathway and guidelines regarding the patients’ hospital admission in this study led to early admission the day before surgery for nine patients in the Magtrace group. Therefore, the iron-based lymph node marking has not led to a reduction in LoS to date. However, educating staff about the new care pathway may lead to lower LoS in the future.

Despite the advantages of Magtrace, some critical aspects need to be mentioned. The rust color of Magtrace serves as a visual aid to lymph node detection,^15^ but it also can impair the aesthetic outcome due to skin discoloration if not applied deep enough.^8^ In most cases, discoloration diminishes over time but may remain for more than 15 months.^8^ In this regard, peritumoral injections seem preferable to reduce skin staining.^29^ In addition, accumulated SPIO has been shown to compromise MRI, which is particularly problematic for patients who need regular breast MRIs, such as patients with hereditary breast cancer.^30^^,^^31^ To address this problem, several studies investigating the feasibility of breast MRI after SPIO application by adjustment of dose, volume, and application mode are currently being conducted.^32^

It should be noted that SPIO can cause allergic reactions similar to blue dye and Tc^99^.^33^^–^35 Consequently, it is crucial to ask patients about allergies, specifically allergies or hypersensitivities against iron oxide or dextran compounds.

Our study was limited by its small sample size, which was calculated with estimated durations of the preoperative course before enrolment. For Tc^99^ use, the estimate was based on our own clinical experience and daily routine. In contrast, the estimation of time expenditure for the Magtrace pathway originated from external colleagues already familiar with the method.

The group allocation was not randomized, which is why selection biases could not be precluded. Furthermore, both patients with breast-conserving surgery and patients with mastectomy were included, limiting the economic analyses. We asked only for pain levels, although in retrospect, detailed patient-reported experience measurements would have been preferable for determination of the patient’s view.

Magtrace is a safe alternative to Tc^99^ for sentinel lymph node localization in breast cancer patients. Its accumulation might be disadvantageous for patients in need of a postoperative breast MRI and could impair the aesthetic outcome due to skin discoloration. However, our study showed that Magtrace shortens the preoperative care pathway and does not affect the reimbursement. In addition, it might lead to an increase in patient comfort as well as a reduction in LoS and surgical time once established. The favorable aspects of Magtrace might particularly benefit patients from rural and underserved areas without a nuclear medicine supply by enabling surgery close to their homes or without multiple appointments at an urban breast center. Ultimately, the new method could facilitate a change in the breast cancer care process toward a shorter and more patient-centered approach.
